# New research frontiers pertaining to the infant gut microbiota 

**DOI:** 10.20517/mrr.2022.12

**Published:** 2022-09-28

**Authors:** Marco Ventura, Douwe van Sinderen, Francesca Turroni

**Affiliations:** ^1^Laboratory of Probiogenomics, Department of Chemistry, Life Sciences and Environmental Sustainability, University of Parma, Parma 43124, Italy.; ^2^Microbiome Research Hub, University of Parma, Parma 43124, Italy.; ^3^APC Microbiome Institute and School of Microbiology, Bioscience Institute, National University of Ireland, Cork T12 YT20, Ireland.

**Keywords:** Microbiome, metagenomics, culturomics, microbe-microbe interactions, host-microbe interactions

## Abstract

The human gut microbiota is believed to be responsible for multiple health-impacting host effects. The influence of gut microorganisms on the human host begins immediately after birth, having long-lasting health effects, while the gut microbiota itself continues to develop throughout the host’s entire life. The purported health-associated effects of the gut microbiota have fueled extensive and ongoing research efforts. Nonetheless, the precise mode of action of functionalities exerted by microbial colonizers of the infant intestine is still largely unknown. The current perspective intends to illustrate major future investigative directions concerning the human gut microbiota with a specific focus on infant-associated gut microbes.

## BACKGROUND

The human gut is considered an organ, most of which is densely colonized by microbial communities, commonly referred to as the gut microbiota^[1]^. The gut microbiota develops and expands during the course of host infancy and adolescence to ultimately reach its relatively stable form during host adulthood^[2-4]^. Increasing scientific and clinical data reveal that the early life gut microbiota elicits, within a critical window of time, long-lasting health effects on its human host^[5-7]^. Remarkably, there are many scientific indications suggesting that specific microbial compositional profiles of the human large intestine are correlated with certain diseases^[8]^. Nevertheless, current knowledge on the composition and associated functions of the intestinal microbiome is mostly derived from bioinformatic analyses, and at best, there are currently fragmented data concerning the biology of intestinal bacteria and the molecular mechanisms that allow microbe-microbe and microbe-host interactions. This thus represents a very serious limitation in defining predictor variables of human health that are based on microbiota composition and their associated functionalities. This knowledge gap is a major impediment preventing clinical advances, as it, for example, would allow us to discern if a microbiota shift observed in an individual represents a cause or a consequence of a disease. Documentation of the factors that drive the establishment and persistence of gut microbiota is still in its infancy. In this context, there is an extensive body of data pertaining to external factors such as mode of delivery, infant feeding mode, gestational age, environments, and prophylactic therapy during pregnancy^[6]^, all of which have been shown to modulate the composition and function of the gut microbiota. Conversely, while the maternal inheritance of bacteria by vertical transmission has been well documented, this being consistent with the co-evolutionary scenario as supported by the holobiont concept^[9,10]^, scientific knowledge on host factors driving such key biological phenomena remains scarce. 

Altogether, this underscores the important scientific challenges that remain to be overcome in terms of elucidating functional aspects exerted by the intestinal microbiome in infants and uncovering host factors responsible for the assembly of the neonatal enteric microbiome. However, despite an ever-increasing interest in the functional discovery of microbiota functionalities, progress in such research endeavors and associated molecular mechanisms is still very slow and patchy at best.

The need to establish and expand our knowledge concerning the functionalities of human gut microbes, therefore, represents a major challenge and research frontier for the microbiologist. Such research efforts may best be initiated with a focus on the infant gut microbiota because it is simpler in terms of the number of bacterial taxa with respect to the adult microbiota, and, as outlined above, infant gut microbes are expected to play a pivotal role in the foundation of human health.

### EXPLORING THE MICROBIOTA DIVERSITY BY CULTUROMICS APPROACHES

**Figure 1:**
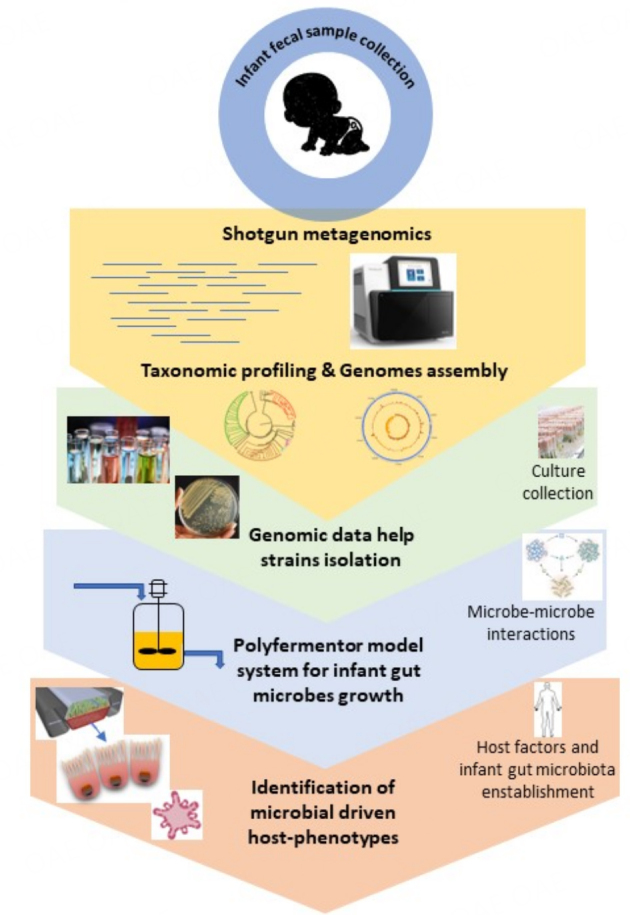
Schematic representation of the models used for the evaluation of the microbe-microbe and host-microbe dialogs. The images here represented are partially adapted from figure of Ventura *et al.*^[35]^.

The establishment of cultivation-independent techniques, in particular 16S rRNA-based microbial profiling and shotgun metagenomics, facilitated the discovery of novel microbes residing in the human body. Most of these newly identified microbes had escaped detection because they were unculturable under the applied *in vitro *conditions^[11-16]^. This prompted the establishment of novel methodologies intended to cultivate these thus far unculturable bacteria [Figure 1]. These approaches consist of high-throughput adaptations and variations of classical growth media for the cultivation of bacteria and standard growth protocols. Thus, interest in these uncultured or not yet cultivated microorganisms encouraged a renaissance of microbiological cultivation approaches^[17]^. Furthermore, the application of various cultivation parameters permitted the identification and subsequent characterization of many new microbial taxa representing elements of this human microbiota dark matter^[11]^. The introduction of culturomics protocols in research projects aimed at exploring as yet unknown elements of the complex intestinal microbiota has provoked an approximately 23% increase in the current collection of cultivated gut microbes^[11]^. MALDI-TOF mass spectrometry allows very reliable and low-cost identification of bacterial isolates in high-throughput culturomics workflows such as those that need to be applied for the precise mapping of the microbial biodiversity of the human gut^[11-16]^. 

The key role of culturomics in the context of dissecting the functional roles played by the enteric microbiome is demonstrated by the fact that the use of this novel omics approach has uncovered the existence of microbial taxa that can be examined under *in vitro* and/or *in vivo* settings to assess their biological features. In addition, culturomics efforts may also produce new therapeutic agents in the form of microbial taxa to be exploited as bacteriotherapy approaches. However, the principal limitation of culturomics is denoted by the considerable requirements of biological material and the difficulty of acting as a high-throughput system (e.g., capable of simultaneously assessing multiple samples). Nevertheless, recent technological developments offer the use of robotic systems which can transform culturomics approaches into high-throughput processing procedures. An additional important constraint of culturomics is represented by the fact that such approaches cannot detect bacteria that were identified by culture-independent attempts, such as 16S rRNA-based microbial profiling, but as yet have escaped isolation, commonly referred to as “still not culturable” bacteria^[11]^. To overcome this limitation, a novel method, i.e., the “hybrid approach”, has recently been established, which is based on the combined use of culturomics trials and deep shotgun metagenomic sequencing^[18]^. Thanks to metabolic modeling facilitated by shotgun metagenomics data, this hybrid approach provides insights into the nutritional requirements and metabolic features of those microbial species that have been recognized as new, thus guiding the design of culturomics approaches for their isolation^[18]^. 

The isolation and subsequent cultivation of those not yet cultivable microorganisms by culturomics approaches will also be important to establish exhaustive public repositories of bacterial strains. In fact, an important new perspective for the future development of gut microbiota-based science is to invest resources that will allow the isolation and subsequent genetic characterization of novel gut microbes representing the complete microbial genetic diversity of the human gut microbiota. In this context, it will also be crucial to archive these gut bacteria as pure culture in the form of viable stocks that can then be stored in international and open-source microbial culture repositories that are publicly available to every scientist interested in investigating the functional contribution of these microorganisms to the human gut. 

### DISENTANGLING HOST-MICROBE INTERACTIONS IN THE HUMAN GUT

As described above, the recent discovery and dissection of the intestinal microbiota in terms of composition and putative correlations with health conditions of the host have facilitated amazing insights, thereby creating therapeutic opportunities. Conversely, very little is known about the functional features of the human gut microbiome and, specifically, about the presumed crosstalk established between enteric bacteria and their human host, i.e., microbe-host communication, as well as with each other, i.e., microbe-microbe interactions [Figure 1]. Importantly, understanding and molecularly dissecting these interactions are considered pivotal to elucidating the functional features provided by each member of the human intestinal microbiome. Consequently, there is an obvious and critical need to explore and disentangle the very complex interactive networks that are believed to exist between enteric bacteria and the human host. Such microbe-host interplays are considered to be crucial in early life when assemblage of the intestinal microbiome is fragile and incomplete, and the host’s metabolism and immune system are still developing and hence exposed to changes originated/driven by microbiome-guided activities. To study such fundamental interactions, a reductionist approach can be applied where both microbial communities and host are set up and presented in a manner to allow easy measurement and flexible adjustment of all possible variables. Novel cutting-edge technologies may offer valuable methodological *in vitro* approaches to perform such experimental trials. In this context, human gut organoids, which have recently been realized as the “gut-on-a-chip” model and represent self-organizing three-dimensional epithelial structures originating from stem cells, are important developments that may be used to mimic the human host^[19-21]^. More specifically, the “gut-on-a-chip” is a microfluidic device in which cells are cultivated with organ-relevant chemical gradients and dynamic mechanical signals, therefore reflecting structural tissue characteristics and functional intricacies of a living organ in an *in vitro* format^[22]^. Furthermore, unlike other host models, such as human cell monolayers, this device is an active arrangement simulating gut peristalsis and containing peripheral blood mononuclear cells and endothelial cells, overall encompassing a highly relevant and valuable human intestinal model. 

Nevertheless, this *in vitro* model system displays some limitations due to the difficulties associated with the settings of the appropriated environmental conditions (e.g., a strictly anoxic environment) for the maintenance of complex microbial communities and the absence of several crucial functionalities of the host, such as the host endocrine system. 

A large part of current scientific literature concerning microbe-host interplays is based on conventional and/or axenic mice^[23]^. Nevertheless, murine models, similar to all other animal models (e.g., rabbit and monkey), have important limitations, which prevent direct translation of animal model-based research results to humans^[24]^. Instead, pig-based models display the potential to be a viable model for host-microbe interactions, particularly for the human infant and gut-brain axis research. 

Synthetic or defined microbial microcosms have recently been described for the purpose of *in vitro* approaches that mimic the human intestine and could be employed to evaluate microbe-microbe crosstalk^[25-27]^. These synthetic microbiota assemblies were accomplished based on complex culturomics approaches, allowing the establishment of defined microbial communities which resemble the adult intestinal microbiome^[28]^. These artificial gut microbiotas are generated to encompass “a core intestinal microbiota”, consistent with the classification of the enteric microbiome in enterotypes^[29]^. The core gut microbiota includes bacterial species, whose biological roles are presumed to be essential for the functionality of the intestinal microbiome^[30]^. Nevertheless, the concepts of enterotypes as well as a core intestinal microbiota are still not universally accepted by the scientific community, and there is still a large debate around them. 

For the purpose of disclosing microbe-microbe crosstalk pathways and mechanisms, it will be crucial to employ *in vitro* models that can host such synthetic microbiotas. In this context, the use of bioreactor systems has been demonstrated to be an efficacious approach to achieving stable cultivation of keystone species of the intestinal microbiota^[31,32]^. Nevertheless, most of these systems do not incorporate the mucosal component of the human gut and, except for some environmental parameters, reproducing those occurring in the human gut, lack the majority of host-produced molecules such as mucins. These limitations are likely to reduce the overall feasibility of these systems in terms of their relevance as models to reproduce microbe-host crosstalk; nonetheless, they may still be valuable to uncover and investigate microbe-microbe interplays. 

### DEEP INSIGHTS INTO THE METABOLIC CONTRIBUTION OF GUT MICROORGANISMS

An important new avenue of research involving the gut microbiota is aimed at understanding the metabolic contribution of gut microbes in infants, with a specific focus on microbial-driven metabolites such as indole-3-lactic acid and its interaction with the host. In this context, it has been shown that tryptophan metabolism by Bifidobacteria leads to the production of indole-3-lactic acid, which in turn affects the host immune system, thus representing an intriguing example of microbe-host communication^[33]^. Similarly, the discovery of other microbiota-derived small molecules, including ribosomal synthetized posttranslational modified peptides, lantibiotics, bacteriocins, microcins, and other products of fatty acid, vitamin, carbohydrate, and/or amino acid metabolism, represent new frontiers of research regarding the functionality of gut microbes and their potential biological role in terms of immune-modulation and antibiosis. Such exploration will be fueled by *in silico* prediction of microbiome data, but it will require a detailed experimental metabolomic validation based on the cultivation of single bacterial strains or synthetic microbiota under *in vitro* settings using gut microbiota models.

### DECIPHERING THE MICROBE-MICROBE INTERACTIONS

To better understand the biological functions of gut microbes, it will be crucial to evaluate how the co-occurrence of different gut microorganisms might influence the molecular dialog of each bacterial strain [Figure 1]. Recently, the *in vitro* reconstruction of the microbiota naturally residing in different human body sites has been achieved using bioreactor models^[28]^. Such an *in vitro* system allows the cultivation and maintenance of a simplified microbiota, i.e., a synthetic microbiota, in an *in vitro* gut environment setting, and, thanks to functional genomics analyses such as metatranscriptomics and metabolomics experiments, it will be possible to identify the microbial genetic elements involved in microbe-microbe crosstalk.

Preliminary analyses involving simple microbial gut communities represented by two bacterial strains cultivated under *in vitro* conditions have delineated trophic interactions aimed at utilizing complex carbon sources (e.g., mucin) that are practically inaccessible to most bacterial strains when cultivated on their own. Such cross-feeding scenarios appear to represent a very common metabolic strategy of members of the human gut microbiota that will most likely target very different classes of molecules, including not only components of the diet or host-produced molecules but also drugs, with very important effects on host physiology and host health.

## CONCLUSIONS

Recently, very significant progress has been made in understanding the composition of the infant intestinal microbiome thanks to the use of novel culture-independent approaches such as metagenomics methods^[34]^. Nevertheless, relatively limited research efforts have focused on investigating microbiome interactions that occur between bacteria and the human host and evaluating how the microbiota composition and its linked metabolic features influence host health. There are fundamental and commercial interests directed at applying microbiome members and/or their metabolic products as accurate human health predictors and developing novel strategies to modify and shape the infant intestinal microbiome, especially in those babies delivered prematurely and/or by Caesarian section (CS) as they frequently possess a gut microbiota that is different from their full-term, vaginally delivered counterparts^[5,7]^. Therefore, future preventive clinical procedures may be aimed at encouraging the establishment of CS/premature babies with an artificial microbiota encompassing bacteria known to be typically obtained from the mother during vaginal birth. 

However, these intervention strategies necessitate a profound knowledge of the composition of the infant intestinal microbiome and the microbiota components responsible for the support of human health. Another important potential application is to artificially influence the establishment of the initial human intestinal microbiome through the pregnant (future) mother. Handling the maternal intestinal microbiome throughout pregnancy is assumed to change the assembly of the gut microbiota during the very early stages of life. Nevertheless, many unanswered questions remain, for example, how and why specific bacterial taxa are acquired by infants as a result of vertical inheritance from their mothers. Furthermore, our knowledge concerning the persistence of certain bacteria in the large intestine of infants upon weaning, as well as their influence on host health conditions, remains relatively limited. Another question that remains open is about the contribution of host genetics in driving the transfer of microbes between generations and the understanding of the genetic contributions shaping intergenerational sharing of microbiomes.

### OUTSTANDING QUESTIONS

-What is the extent of the microbial biodiversity residing in the human gut? 

-What is the functional contribution of the gut microbes to the host’s wellness?

-Are there bacterial strains that persist in the human gut for the entire life of a human being?

-How should gut microbes be selected to constitute a synthetic microbiota?

-What is the contribution of host genetics in modulating the vertical transmission of microbes from one human generation to the next? 

## DECLARATIONS

### Copyright

© The Author(s) 2022
